# Maternal Diet Influences Fetal Growth but Not Fetal Kidney Volume in an Australian Indigenous Pregnancy Cohort

**DOI:** 10.3390/nu13020569

**Published:** 2021-02-09

**Authors:** Yu Qi Lee, Eugenie R Lumbers, Tracy L Schumacher, Clare E Collins, Kym M Rae, Kirsty G Pringle, Gomeroi gaaynggal Advisory Committee

**Affiliations:** 1 Saw Swee Hock School of Public Health, National University of Singapore, Singapore 117549, Singapore; ephlyq@nus.edu.sg; 2 Pregnancy and Reproduction Program, Hunter Medical Research Institute, New Lambton, NSW 2305, Australia; Eugenie.Lumbers@newcastle.edu.au; 3 Priority Research Centre for Reproductive Sciences and School of Biomedical Science and Pharmacy, University of Newcastle, Callaghan, NSW 2308, Australia; 4 Priority Research Centre for Physical Activity and Nutrition, University of Newcastle, Callaghan, NSW 2308, Australia; tracy.schumacher@newcastle.edu.au (T.L.S.); clare.collins@newcastle.edu.au (C.E.C.); 5 Department of Rural Health, University of Newcastle, Tamworth, NSW 2340, Australia; 6 Priority Research Centre for Health Behaviours, University of Newcastle, Callaghan, NSW 2308, Australia; 7 School of Health Sciences, College of Health, Medicine and Wellbeing, University of Newcastle, Callaghan, NSW 2308, Australia; 8 Mater Medical Research Institute, South Brisbane, QLD 4101, Australia; kym.rae@mater.uq.edu.au; 9 Faculty of Medicine, University of Queensland, Herston, Brisbane, QLD 4072, Australia

**Keywords:** pregnancy, nutrition, Indigenous, fetal growth, kidney

## Abstract

Suboptimal nutrition during pregnancy is recognised as a significant modifiable determinant in the development of chronic disease in offspring in later life. The current study aimed: (i) to assess the dietary intakes of pregnant Indigenous Australian women against national recommendations and (ii) to investigate the associations between maternal nutrition during pregnancy and the growth of the offspring, including kidney development in late gestation in the *Gomeroi gaaynggal* cohort (*n* = 103). Maternal dietary intake in the third trimester was assessed using the Australian Eating Survey Food Frequency Questionnaire. Estimated fetal weight (EFW) and kidney size were obtained by ultrasound. Birth weight was retrieved from hospital birth records. Of the five key nutrients for optimal reproductive health (folate, iron, calcium, zinc and fibre), the nutrients with the highest percentage of pregnant women achieving the nutrient reference values (NRVs) were zinc (75.7%) and folate (57.3%), whereas iron was the lowest. Only four people achieved all NRVs (folate, iron, calcium, zinc and fibre) important in pregnancy. Sodium and saturated fat intake exceeded recommended levels and diet quality was low, with a median score of 28 out of 73 points. After adjusting for smoking and pre-pregnancy body mass index, only maternal intake of retinol equivalents and the proportion of energy from nutrient-dense or energy-dense, nutrient-poor (EDNP) foods were associated with fetal growth. EFW decreased by 0.13 g and birth weight decreased by 0.24 g for every µg increase in maternal dietary retinol intake. Interestingly, EFW, but not actual birth weight, was positively associated with percentage energy from nutrient dense foods and negatively associated with percentage energy from EDNP foods. Dietary supplement usage was associated with increased birthweight, most significantly iron and folate supplementation. Current dietary intakes of pregnant Australian women from this cohort do not align with national guidelines. Furthermore, current findings show that maternal retinol intake and diet composition during pregnancy can influence fetal growth, but not fetal kidney growth in late gestation. Strategies that aim to support and optimise nutrient intakes of Indigenous pregnant women are urgently needed. Future studies with long-term follow-up of the children in the current cohort to assess renal damage and blood pressure are imperative.

## 1. Introduction

Maternal dietary intake influences the supply of nutrients required for optimal fetal growth, and so potentially has life-long impacts for offspring’s future health [1,2]. Events such as the Dutch Famine of 1844–1945 [3] highlighted the association between malnutrition during pregnancy and birth weight. Nutrient inadequacies during pregnancy can impair fetal growth, which can in turn increase risk for low birth weight (LBW), small-for-gestational-age (SGA) or preterm delivery [4,5,6] or alterations in infant body composition [7]. Infants born with LBW have a greater risk of developing hypertension, cardiovascular disease and diabetes mellitus in middle age [8,9,10,11,12].

The developing kidney is particularly vulnerable to the effects of a suboptimal maternal diet. Numerous studies in animals have demonstrated that maternal over- or under-nutrition can lead to altered nephrogenesis, with a permanent reduction in nephron endowment leading to detrimental consequences for offspring renal health [13,14]. Maternal protein restriction in pregnancy is associated with reduced birth weight, impaired nephron endowment, elevated blood pressure and reduced glomerular filtration rate (GFR) in offspring across a variety of animal models [15,16,17,18]. There is also some evidence that deficiencies in maternal folate, vitamin A, and total energy intake during pregnancy are associated with detrimental impacts on fetal kidney volume, a surrogate measure for nephron number [13,19]. While there is convincing evidence from animal studies that maternal nutrition impacts kidney development, evidence from human studies is not as strong [20], highlighting the need for more work in this area. 

Australia’s Aboriginal and Torres Strait Islander populations (hereafter respectfully termed Indigenous) are experiencing an epidemic of chronic disease, particularly diabetes, renal disease and chronic renal failure [21,22]. Indigenous Australians are twice as likely as non-Indigenous Australians to develop chronic kidney disease (CKD) [22,23], contributing significantly to the disparity in life expectancy between Indigenous and non-Indigenous Australians [22,24]. It has been suggested that the higher prevalence of LBW and/or preterm births among Indigenous people, may be partly responsible for the greater prevalence of CKD and end stage kidney disease [25]. Nephron endowment in Indigenous Australians is significantly reduced compared to non-Indigenous people [26] and Indigenous Australian babies of LBW were reported to have fewer nephron numbers and larger glomerular volumes [27,28]. Since LBW and intrauterine growth restriction are associated with a reduced kidney volume, an indicator of nephron number, this could be one of the mechanisms via which programming of kidney disease occurs [29]. 

There is a paucity of research assessing the dietary intake of Indigenous women in Australia during pregnancy. Previously, we have reported that a small cohort of women from the *Gomeroi gaaynggal* study (*n* = 58) had dietary intakes that did not meet the recommendations outlined in the Australian Dietary Guidelines for pregnancy [30]. However, to date, the impact of maternal nutrition during pregnancy on fetal growth and kidney development in utero in the *Gomeroi gaaynggal* cohort has not yet been fully explored. Accordingly, the present study aimed: (i) to assess whether the dietary intakes of pregnant Indigenous Australian women from a larger cohort (*n* = 103) of the *Gomeroi gaaynggal* study align with national dietary guidelines; (ii) to determine, in an Indigenous Australian population, potential associations between maternal nutrition during pregnancy and the growth of the offspring, including kidney development in late gestation.

## 2. Materials and Methods 

### 2.1. Setting 

This study used data collected from the *Gomeroi gaaynggal* study, a prospective longitudinal cohort of Indigenous Australian mother–child dyads followed from pregnancy, through the postnatal period and up until the children are 10 years of age. This study will only present and discuss data from the pregnancy study. The primary site of the study was Tamworth, a rural town in New South Wales, Australia. Further details of the *Gomeroi gaaynggal* study have been published elsewhere [31].

### 2.2. Ethics

Ethical approval for the study was obtained from the following committees: Hunter New England Human Research Ethics Committee (HNEHREC No. 08/05/21/4.01); the New South Wales Human Research Ethics Committee (NSW HREC HREC/08/HNE/129); and the Aboriginal Health and Medical Research Council Human Research Ethics Committee (AHMRC HREC 654/08).

### 2.3. Recruitment

Recruitment began in 2010 and ended in May 2019 with eligible pregnant women recruited by Indigenous research assistants who attend antenatal clinics at Tamworth Rural Referral Hospital (*n* = 370). Pregnant women who identified as Indigenous Australian, or non-Indigenous pregnant women with Indigenous partners were eligible to participate and could enrol at any stage in their pregnancy. Written informed consent was required to participate in the study.

### 2.4. Study Design 

The aim was to carry out antenatal visits once every trimester at <13, 13–28 and >28 weeks of gestation. At each visit, a range of assessments were undertaken including questionnaires, physical examinations, biological sample collection (maternal blood and urine), and fetal ultrasounds. Details regarding data collected at each time point have been published elsewhere [31]. 

Gestational age was determined by ultrasound at the first study visit. Additionally, at this visit, information on maternal age at recruitment, Indigenous status of both parents, maternal educational level, cigarette smoking during pregnancy, pre-pregnancy weight, obstetric history, and other pre-existing medical conditions, including diabetes, asthma, hypertension, and kidney disease were collected via a self-reported questionnaire.

### 2.5. Maternal Anthropometry

Maternal pre-pregnancy body mass index (BMI) was calculated from measured height (ht) and self-reported pre-pregnancy weight (wt) at their first visit during pregnancy [wt(kg)/ht(m^2^)] and each participant was subsequently categorised as being underweight (BMI < 18.5 kg/m^2^), normal weight (BMI 18.5–24.9 kg/m^2^), overweight (BMI 25.0–29.9 kg/m^2^) or obese (BMI ≥ 30.0 kg/m^2^) according to World Health Organization definitions [32]. Maternal height was measured without shoes to the nearest 0.1 cm using a wall-mounted stadiometer and a headboard (Model 0123; Seca, Germany) with the head positioned in the Frankfort plane. For participants who could not recall their pre-pregnancy weight, pre-pregnancy BMI was calculated using their current weight providing it could be measured prior to 12 weeks of gestation.

### 2.6. Maternal Dietary Assessment during Pregnancy 

During the third trimester, maternal dietary intake was assessed using the Australian Eating Survey Food Frequency Questionnaire (AES FFQ) [33]. The AES FFQ is a self-administered 120-item semi-quantitative FFQ that asks respondents to report their usual dietary intake over the previous 6 months [33], with frequency options ranging from ‘never’ to ‘≥7 times per day’. Standard portion sizes were determined for each food item using data derived from the most current National Nutrition Survey [34]. An example of a ‘natural’ serving size for standard items would be a slice of bread. This survey includes 15 supplementary questions on age, use of vitamin supplements, food behaviours and sedentary behaviours. 

Nutrient intakes from the FFQ were computed from the AUStralian Food and NUTrient (AUSNUT) 2011–13 database (all foods). This survey has been shown to provide a valid and reliable estimate of usual dietary intake of Australian adults over the previous 6 months [33] and also fruit and vegetable intakes in women relative to plasma carotenoid concentrations [35]. Further details of the AES FFQ are provided elsewhere [33].

### 2.7. Australian Guide to Healthy Eating 

The 2013 Australian Guide to Healthy Eating (AGHE) recommends specific numbers of daily serves of five core food groups based on age and sex that are consistent with meeting key nutrient reference values (NRVs) [36]. The nutrient-dense or core food groups include breads/cereals (grains), lean meat and vegetarian alternatives (including eggs, nuts and legumes), vegetables (including legumes), and fruit and dairy [36]. Non-core foods are energy-dense, nutrient-poor (EDNP) or ‘junk’ foods that are not a necessary part of a healthy diet and are recommended to be consumed in limited amounts and less frequently than core foods [36].

### 2.8. Nutrient Reference Values (NRVs)

Specific daily nutrient intake targets are recommended by the 2006 National Health and Medical Research Council of Australia to optimize health and avoid nutritional deficiency [37]. The estimated average requirement (EAR) and adequate intake (AI) are the most appropriate NRVs for comparison with population group intakes and the focus of the present study.

### 2.9. Australian Recommended Food Score (ARFS)

Diet quality scores were calculated using the Australian Recommended Food Score (ARFS) [38], which utilises a subset of 70 AES FFQ questions where the regular consumption of an item aligns with recommendations in the 2013 Australian Dietary Guidelines, as described in detail elsewhere [38]. This diet quality score was previously validated using plasma carotenoids [39]. The ARFS has a total possible score between the range of zero and 73. This includes eight food group subscales: vegetables (0–21), fruit (0–12), meat (0–7), meat alternatives (0–6), grain (0–13), dairy (0–11), water (0–1) and condiments (0–2). Briefly, points are awarded for food consumed according to frequency of consumption, with healthy foods receiving bonus points. The ARFS score was calculated by adding the points for each item. A higher score indicates greater alignment with the Australian National Dietary Guidelines [36].

### 2.10. Ultrasound Measurements 

#### 2.10.1. Fetal Biometry

Fetal biometrics and kidney measurements were measured at the same visit. Ultrasound examinations were performed using a Phillips Cx50 Portable Diagnostic Ultrasound with a 5 MHz convex transducer, which was used to determine gestational age and fetal measurements. Fetal biometry including head circumference, abdominal circumference, and femur length. Estimated fetal weight (EFW) was calculated using the formula by Hadlock using head circumference (HC), abdominal circumference (AC) and femur length (FL): EFW (g) = 10.9 × (1.326 − 0.00326 × AC × FL + 0.0107 × HC + 0.0438 × AC + 0.158 × FL) [40].

#### 2.10.2. Fetal Kidney Measurements

Kidney length, anterior–posterior diameter (thickness) and transverse diameter (width) were determined by ultrasound. Renal length was the maximum longitudinal length measured. Anterior–posterior diameter was measured as the maximum distance between the anterior and posterior wall of the kidney. The transverse diameter was the maximum transverse diameter on a transverse scan. Anterior–posterior and transverse kidney diameter were measured perpendicular to each other, outer to outer, above the hilum [41]. Kidney volume was calculated using the formula for an ellipsoid: volume (cm^3^) = length (mm) × transverse (mm) × anterior–posterior (mm) × 0.523) [42]. Combined kidney volume (cm^3^) was calculated as the sum of the left and right kidney volume. Relative combined kidney volume was calculated as the ratio of combined kidney volume/EFW (cm^3^/kg). Relative kidney measurements better represent kidney size than absolute kidney measurements as it eliminates sex and length differences [43,44]. For this study, only the kidney measurements taken in the third trimester were used in the analysis.

#### 2.10.3. Pregnancy Outcome Measures

Maternal pregnancy and birth outcomes including birth weight, infant sex, gestational age at delivery (days) and maternal and neonatal problems such as gestational diabetes (GDM) (diagnosed by a fasting glucose of ≥5.5 mmol/L), preeclampsia, gestational hypertension, prematurity and LBW were obtained from maternal hospital records. Guidelines for the diagnosis of preeclampsia and gestational hypertension were obtained from the International Society for the Study of Hypertension in Pregnancy (ISSHP) [45].

#### 2.10.4. Population for Analysis 

From the cohort dataset, only those with complete data for both the third trimester kidney ultrasound and AES FFQ were included in the current analysis (*n* = 108). Twin pregnancies (*n* = 1) were excluded. Participants with extreme Vitamin A intake levels (>6000 ug, *n* = 4) were excluded, with the remaining 103 mothers included in the final sample.

#### 2.10.5. Statistical Analysis

Continuous data were summarised using descriptive statistics and were tested for normality, with normally distributed data reported as mean (95% confidence interval (CI)) and non-normal data reported as median [interquartile range (IQR)]. Categorical data were summarized as counts and percentages of each category. Multiple linear regression models were used to assess the association between maternal diet during pregnancy and fetal kidney volume and EFW in the third trimester of pregnancy, and birth weight. A directed acyclic graph (DAG) was used to visually select variables to be adjusted for. A priori content knowledge was used in this process as well. The model focusing on the fetal kidney volume was adjusted for EFW and smoking. The model focusing on the relative fetal kidney volume was adjusted for smoking only. The model focusing on EFW and birth weight was adjusted for smoking, gestational age in days (at ultrasound or at delivery) and pre-pregnancy BMI. 

Additionally, multiple linear regression models with adjustments were used to investigate associations between dietary nutrient intake, including supplement intake, with EFW, birth weight and fetal kidney volume. Additional median intake of nutrients (calcium, folate, iron and zinc) from vitamin supplements were imputed for those who answered ‘yes’ to the FFQ question regarding use of vitamin supplements during pregnancy (*n* = 50/103). The median intake of these key nutrients from supplements was obtained from 152 pregnant women from the *Gomeroi gaaynggal* study using 24 h recalls completed in the first trimester [46]. 

## 3. Results

### 3.1. Participant Characteristics

The baseline characteristics of the women in this Indigenous cohort with singleton pregnancies who were included in this analysis are summarized in Table 1. The median maternal age at the time of consent was 23.9 years (interquartile range (IQR): 21.1, 29.3, *n* = 103). Of those who self-reported their pre-pregnancy weight, 5.2% (*n* = 5/96) of mothers were underweight (BMI < 18.5 kg/m^2^), 27.1% (*n* = 26/96) were within the normal weight range (BMI 18.5–24.99 kg/m^2^) and 67.7% (*n* = 65/96) were overweight/obese (BMI ≥ 25.0 kg/m^2^); 30.3% (*n* = 30/99) of the women in the cohort reported smoking during pregnancy. 1.9% of women in the cohort (*n* = 2/103) had pre-existing type 1 diabetes, none had diagnosed pre-existing type 2 diabetes, 10.7% (*n* = 11/103) developed GDM, none of the women had chronic hypertension, 1.9% (*n* = 2/103) had gestational hypertension and 4.9% (*n* = 5/103) developed preeclampsia during their pregnancy. The majority of the women had a high school education or less (70.4%). 

Both maternal dietary intake and fetal kidney ultrasound were measured at a mean gestational age of 35.1 weeks. Of those with fetal sex information available (*n* = 97), 36 babies were female (37%) and 61 were male (63%). Of those with birth outcome data available (*n* = 93), 90 babies (97%) were born at term (>37 weeks) with a median age at birth of 39.1 weeks (IQR: 38.4, 40.2) and a median birth weight of 3.47 kg (IQR: 3.06, 3.79). Three were born prematurely (3%), with a median age at birth of 36.5 weeks (min, max: 36.4, 37.2) and a median birth weight of 3.41 kg (IQR: 3.38, 3.67).

### 3.2. Maternal Dietary Intakes

Table 2 summarises the participant dietary intakes of selected nutrients from food sources alone, including total energy, macronutrients, selected micronutrients and comparisons with NRVs. Median intakes of protein, zinc, thiamin, riboflavin, niacin, vitamin C, potassium, magnesium, phosphorus were above the estimated pregnancy EAR or AI. Folate, iron, calcium, zinc and fibre are considered the most important for optimal reproductive health [47,48,49,50]. Of these, the nutrients with the highest number of women meeting the recommendations were zinc (75.7%; *n* = 78/103) and folate (57.3%; *n* = 59/103). Half of the women achieved the calcium recommendation whereas iron had the lowest adequacy rate (3.88%; *n* = 4/103). Only four of the participant’s dietary intakes met all pregnancy NRVs (folate, iron, calcium, zinc, fibre). The median vitamin A (retinol equivalents) intake was twice the EAR and three participants had an intake above the upper limit of 3000 μg/day. The median sodium intake was twice the upper end level of estimated AI. The median percentage of energy contributed from saturated fat was 15%, which is above the maximum recommended level of 10% [37]. 59% of energy was derived from nutrient-dense foods while 41% of energy was derived from energy-dense, nutrient-poor (EDNP) foods (Table 3). Total ARFS and subscale diet quality scores are summarised in Table 3. This cohort obtained a median score of 28, out of the maximum possible score of 73, indicative of low diet quality. The vegetable, fruits, grains and meat and alternatives subscale scored the lowest relative to the total number of points available in each component.

### 3.3. Associations between Maternal Dietary Intake and Estimated Fetal Weight (EFW), Birth Weight and Fetal Kidney Volumes 

We assessed whether there were any associations between maternal dietary intake from food alone during pregnancy and EFW and birth weight, as well as third trimester fetal kidney volume. Micronutrients of particular interest were retinol, folate, iron, sodium, calcium and zinc (Table 4). Macronutrients of particular interest were protein, carbohydrate, protein:carbohydrate and energy (percentage from nutrient-dense and EDNP foods) (Table 4). Association with diet quality is also presented in Table 4. Associations for other nutrients are shown in Supplementary Tables S1 and S2. 

After adjusting for smoking, gestational age in days (at ultrasound scan) and pre-pregnancy BMI, maternal dietary retinol intake was negatively associated with EFW. EFW decreased by 0.13 g for every µg increase in maternal dietary retinol intake. The percentage energy intake from EDNP foods was negatively associated with EFW. EFW decreased by 5 g for every percent increase in percentage energy intake from EDNP foods. In contrast, percentage energy intake from nutrient-dense foods was positively associated with EFW. EFW increased by 5 g for every percent increase in percentage energy intake from nutrient-dense foods. Any other micronutrients, macronutrients or diet quality were not associated with EFW (Table 4 and Supplementary Table S1). As unadjusted regression models for these outcomes were similarly statistically significant, only the adjusted models are reported here. 

After adjusting for smoking, gestation age in days (at delivery) and pre-pregnancy BMI, maternal dietary retinol intake was negatively associated with birth weight (kg). Birth weight decreased by 0.24 g for every µg increase in maternal dietary retinol intake. Any other micronutrients, macronutrients or diet quality were not associated with birth weight (Table 4 and Supplementary Table S1). As unadjusted regression models for these outcomes were similarly statistically significant, only the adjusted models are reported here. 

Regardless of whether the regression model was adjusted or unadjusted, none of the micronutrients or macronutrients of interest, diet quality (Table 5), or other nutrients (Supplementary Table S2) were associated with combined kidney volume (cm^3^) and combined kidney volume relative to EFW (cm^3^/kg).

### 3.4. Effect of Dietary Intake and Vitamin Supplementation on EFW, Birth Weight and Fetal Kidney Volumes

Associations between maternal calcium, folate, iron and zinc intake from food and supplements during pregnancy and EFW and birth weight, as well as third trimester fetal kidney volume are presented in Table 6 and Table 7. After adjusting for smoking, gestation age in days (at delivery) and pre-pregnancy BMI, maternal folate and iron intake were positively associated with birth weight (kg). Birth weight increased by 0.33 g for every µg increase in maternal folate intake. Additionally, birth weight increased by 7.0 g for every mg increase in maternal iron intake. None of the other nutrients were associated with EFW or fetal kidney volume. As unadjusted regression models for these outcomes were similarly statistically significant, only the adjusted models are reported here. 

## 4. Discussion

There is increasing evidence indicating that maternal dietary intake and nutritional status during pregnancy not only influences pregnancy outcome but has long-term consequences for maternal health and offspring health throughout life [51,52,53,54,55,56]. To the best of our knowledge, this is the first study that examines the possible association between maternal dietary intake during pregnancy, human fetal growth and kidney growth in an Indigenous Australian population residing in a rural region of New South Wales, Australia. Overall, the current analysis shows that pregnant Indigenous women may benefit from a higher diet quality and that targeting the intakes of key micronutrients important for perinatal and long-term child health would be of value. EFW was positively associated with the percentage of total energy intake from nutrient-dense foods and negatively associated with percentage of energy from energy-dense, nutrient-poor (EDNP) foods. Maternal dietary intake of vitamin A was negatively associated with fetal growth and birth weight. Additionally, folate and iron dietary supplement usage was associated with increased birthweight. No association was found between maternal dietary intake during pregnancy and fetal kidney volume in late gestation. 

To date, very limited existing literature reports the dietary intakes of Indigenous women of childbearing age as well as during pregnancy. Intakes of fruits and vegetables in this cohort of pregnant Indigenous women appears to be suboptimal. Intakes of saturated fat and sodium during pregnancy were above the recommended levels for the majority of the mothers. Similarly, in a cross-sectional survey of young Indigenous women (*n* = 424 Aboriginal, *n* = 232 Torres Strait Islander) of childbearing age in rural Queensland [57], self-reported fruit and vegetable consumption was low, with only 12 women (<2%) reported to have met the dietary recommendations for fruit and vegetable intake (defined as ≥2 fruit and ≥5 vegetables). These findings are consistent with a recent systematic review [58], which found that Indigenous people in Australia consume too little of the five major food groups, with excessive consumption of EDNP food. Sodium intake above the recommended level has also been reported in a remote Indigenous Australian general population, where 46% of all sodium intake was contributed by discretionary salt, bread and processed meat [59]. Findings were similar in a number of studies conducted in the wider population of pregnant women from Australia [60,61,62,63,64], the United States of America [65] and the United Kingdom [66]. There are potential health consequences associated with a high-fat, high-sodium diet, particularly in highly processed foods during pregnancy, including altered placental function and predisposition to metabolic disease in the offspring [67]. There is no doubt that having an eating pattern that aligns with the dietary guidelines is challenging for many pregnant women, both Indigenous and non-Indigenous alike [62,68]. This highlights the fact that pregnant women need more support to improve their dietary intake in order to optimise nutrient intakes.

Only four women (3.88%) met the NRVs for all five key nutrients important in pregnancy (fibre, calcium, iron, folate and zinc) from food sources alone. These data indicate a low conformance with NRVs among pregnant Indigenous Australian women from the *Gomeroi gaaynggal* cohort. Similar results were reported in a smaller group of 58 mothers from the *Gomeroi gaaynggal* cohort with a similar sociodemographic where only one woman achieved the NRVs for all key nutrients (folate, iron, calcium, zinc, fibre) from food sources alone [30]. It has similarly been found that dietary intakes of folate, iron and vitamin D were consistently below nutrient recommendations in pregnant women from developed countries [49], as well as low- and middle-income countries [69]. This supports the universality of inadequate intakes of important nutrients such as folate and iron among pregnant women world-wide, and the difficulties women face in meeting increased requirements during pregnancy from food sources alone. They may not be aware of the need to increase important nutrients [70]. This emphasises the need to supplement the diet with additional nutrients, for example through the fortification of food products and the recommendation of routine prenatal supplementation. 

The current study found that maternal dietary intake of vitamin A was negatively associated with fetal growth and birth weight. Similar results were reported in a prospective cohort study of 439 European and Polynesian pregnant women where high β-carotene and retinol intakes were associated with decreased birthweight [71]. Diet was assessed using a 24 h recall and a 3-day food record [71]. This is in accordance with a recent large prospective study showing that maternal serum vitamin A concentrations during pregnancy were positively associated with LBW, and negatively associated with macrosomia [72]. Conversely, a recent systematic review and meta-analysis of 17 trials reported that vitamin A or beta-carotene levels during pregnancy did not have a significant overall effect on birthweight indicators, preterm birth, stillbirth, miscarriage or fetal loss [73]. The existing significant disparity in terms of methods used to evaluate maternal vitamin A level during pregnancy, either dietary intake or serum level, sample sizes and methods of measuring the study variables could be reasons for the contradictory findings. This implies the need for studies with standardised measurement of vitamin A levels, adequate design, standardised methods of measuring the study variables, and similar sample size to assess the possible contribution of vitamin A status to birth outcome. Vitamin A is required for maintaining adequate growth and development [73] and maintaining a moderate concentration of vitamin A during pregnancy might be beneficial to achieve optimal fetal growth and birth weight. 

Interestingly, EFW was positively associated with percentage energy intake from nutrient-dense foods and negatively associated with percentage energy intake from EDNP foods. A similar relationship can be observed for birthweight, but results were not statistically significant. This is perhaps not surprising given that current evidence suggests that maternal diet can influence fetal growth [74,75]. In a recent systematic review and meta-analysis of the relationship between maternal dietary patterns during pregnancy and risk of preterm birth and birth weight, it was found that unhealthy dietary patterns during pregnancy, characterized by high intake of refined grains, processed meat, and foods high in saturated fat or sugar, were associated with lower birth weight (mean difference: −40 g; 95% confidence interval: −61, −20 g; I2 = 0%) and a higher risk of preterm birth (odds ratio: 1.17; 95% confidence interval: 0.99, 1.39; I2 = 76%) [76]. Furthermore, another systematic review by Gete et al. has found that a high consumption of vegetables, fruits, legumes, seafood/fish and milk products was associated with a lower risk of SGA [77]. Together, this suggests that improving the composition of the diet in pregnant women, i.e., a higher proportion of the daily energy intake from nutrient dense foods, including vegetables, fruits, wholegrains, low-fat dairy, and lean proteins, could improve fetal growth and go some way to reducing the high rates of LBW babies born in Indigenous Australian populations, as well as improving the long-term health of the children [78]. 

Our results did not find any association between diet quality, which is a measure of variety of nutrient dense foods, and fetal growth or birthweight. This is similar to the findings from the Growing Up in Singapore Towards Healthy Outcomes Study [79] and the Infant Feeding Practices Study II [80]. However, few other studies have shown that higher adherence to a good quality diet during pregnancy is associated with lower risk of having a low birthweight baby [81,82,83]. These inconsistent results between studies can be attributed to the use of different diet-quality scores. These diet-quality indices differ widely with regard to the number of food groups included, cut-off values, and scoring systems according to country-specific national dietary guidelines and contributions of specific components to the overall score [84,85]. More research regarding the association between diet quality and birth outcomes is warranted. 

The current study did find that folate and iron dietary supplement usage was associated with increased birthweight. Our findings are consistent with other prospective cohort studies conducted in rural areas of New Zealand [71] and India [86]. Similarly, two double-blind, randomized controlled trials conducted among different populations of pregnant women in the United States demonstrated a positive effect of iron supplementation on birth weight [87]. In rural Nepal, a cluster-randomized, controlled trial showed that iron and folic acid supplementation resulted in a significant reduction in LBW [88]. A systematic review was suggestive of a positive effect of iron supplementation on birth weight [89]. The evidence of a beneficial effect of maternal iron and folate supplementation is the most convincing in the current literature. Improving access to, and compliance with iron and folate supplementation during pregnancy is needed to improve maternal nutrition during pregnancy and may help to reduce the high rates of LBW in the Indigenous Australian population. 

It is important to note that even though statistically significant associations were found between maternal dietary intake of vitamin A, folate, and iron with fetal growth in late gestation and birth weight in our study, the absolute changes are minimal and may not be clinically relevant. Further studies with larger sample sizes are required to provide guidance for future policy implementation. 

Here, we show that the maternal dietary intake of pregnant women in the *Gomeroi gaaynggal* cohort does not significantly influence offspring kidney size in utero. To the best of our knowledge, this is the first human study to examine associations between maternal nutrient intake and fetal kidney growth during pregnancy. While this data suggests that the nutrient intake of women in the cohort is sufficient to support adequate kidney development, at least in utero, there are several reasons why we may not be seeing any influences of maternal diet during pregnancy on fetal kidney volume in late gestation. Firstly, the current analysis may be premature, before any effects become apparent and, secondly, the nutrient intakes of pregnant women in the current study may not be suboptimal to the degree that it influences fetal kidney development. From our recent systematic review of human studies [20], several key nutrients were found to be of importance for kidney size and function in offspring and the time of kidney assessment in offspring ranged from 3 days old to 50 years of age. Hawkesworth et al. have shown that estimated GFR was significantly higher in children aged 4–5 years whose mothers received 60 mg vs. 30 mg iron during pregnancy but there was no effect on kidney volume [90]. Similar to iron, previous studies have shown that maternal total protein intake is associated with higher estimated GFR but not kidney volume [91] in school-aged children. No studies examined offspring kidney size in late gestation. Further studies in humans are warranted to confirm the association between maternal diet during pregnancy and fetal kidney development in late gestation. Furthermore, although we did not find any association between maternal dietary intake and kidney volume in utero, longer-term follow up studies in this population are required to investigate whether maternal dietary intake during pregnancy influences offspring kidney function in the long-term. 

The strengths of this study include: (1) this is the first study examining the association between dietary intake of pregnant Indigenous Australian women and offspring kidney development in late gestation and birth outcomes; (2) the detailed analysis of renal ultrasound examinations; (3) collection of data and clinical information from patient hospital records; (4) utilising a reliable, valid and useful instrument such as the FFQ to assess habitual dietary intake during pregnancy; and (5) larger sample size compared to the previous study to analyse maternal dietary intake. 

This study has several limitations. We were only able to include participants with matched dietary and ultrasound data that had been collected in the third trimester of pregnancy. Although >350 women have participated in the *Gomeroi gaaynggal* cohort, to date, the collection of dietary data did not begin until well after the study began and, due to the length of time required for study visits, collection of all data for each visit is difficult to obtain due to time constraints for the women and their families. Future research with the *Gomeroi gaaynggal* community thus aims to reduce the number and duration of visits to decrease this time burden on women and their families while maintaining the quality of the study. Another limitation is that pre-pregnancy weight was self-reported. However, previous studies have shown that self-reported pre-pregnancy weight is reliable and valid to be used to estimate pre-pregnancy BMI and gestational weight gain (GWG) [92,93]. Details regarding types of supplementation during pregnancy was not included as part of the AES FFQ and it was known that our cohort of pregnant women from the *Gomeroi gaaynggal* study are taking them, mostly for iron, zinc and folate. Also, the FFQ did not capture added salt at the table or during cooking. 

## 5. Conclusions

Taken together, our data suggest that the dietary intakes of pregnant Indigenous Australian women from the *Gomeroi gaaynggal* cohort do not align with all national recommendations for pregnancy. Key areas for intervention that may be considered by Indigenous communities include low intakes of fruits, vegetables, whole grains, and excess sodium and saturated fat consumption. Identifying aspects of pregnancy dietary intakes that differ from national nutrient recommendations may assist with the development of targeted nutrition interventions and inform public health policies seeking to optimise maternal nutrition, particularly in vulnerable populations. Nutrition knowledge in pregnant women was found to be positively associated with age, household income, and education level [94,95]. Hence, there is a need for more culturally relevant and targeted dietary interventions that focus on optimising diet quality in high-risk groups such as the pregnant Indigenous Australian women. The inclusion of the Indigenous community, awareness of social determinants and the local context in developing sustainable nutrition interventions are essential components for success [96,97,98].

Additionally, this study is the first to investigate the association between maternal dietary intake during pregnancy and fetal kidney volume during late gestation in an Indigenous Australian population. Although maternal dietary intake during pregnancy was found to have no influence on fetal kidney development in late gestation, future studies with long-term follow-up of the children in the current cohort to assess renal damage and blood pressure are imperative to determine whether, and to what extent, maternal dietary intake during pregnancy influences offspring renal function. 

## Figures and Tables

**Table 1:** Sociodemographic characteristics of pregnant women in the *Gomeroi gaaynggal* cohort (*n* = 103).

Variables	*n* (%)
Indigenous Status (*n* = 103)	
Indigenous	81 (78.6)
Carrying an Indigenous child	22 (21.4)
Educational level (school attainment) (*n* = 81)	
<Year 10	8 (9.9)
Year 10 or equivalent	28 (34.6)
Year 12 or equivalent	21 (25.9)
Trade/apprenticeship	14 (17.3)
Undergraduate degree	5 (6.2)
Post-graduate degree	2 (2.5)
Currently studying	3 (3.7)
Pre-pregnancy BMI status (kg/m^2^) (*n* = 96)	
Underweight (<18.5 kg/m^2^)	5 (5.2)
Normal weight (18.5–24.99 kg/m^2^)	26 (27.1)
Overweight/obese (≥25.0 kg/m^2^)	65 (67.7)
Number with diabetes mellitus (*n* = 103)	
Type 1	2 (1.9)
Type 2	0
Gestational diabetes	11 (10.7)
Number with hypertensive disorders (*n* = 103)	
Chronic hypertension	0
Gestational hypertension	2 (1.9)
Preeclampsia	5 (4.9)
Smoked during pregnancy (*n* = 99)	
Yes (at any point during pregnancy)	30 (30.3)

BMI: Body Mass Index.

**Table 2:** Daily dietary intakes of selected nutrients in pregnant Indigenous women from the *Gomeroi gaaynggal* cohort (*n* = 103).

Meeting	EAR	Median	IQR	Meeting EAR or AI *n* (%)
Energy (kJ/day)	-	8506	6918–11434	-
Protein (g)	47 (≤18 y) 49	92.0	69.6–122.4	95 (92.2)
Fibre (g)	25 * (≤18 y) 28 *	23.0	15.9–30.4	37 (35.9)
Thiamin (mg)	1.2	1.6	1.1–2.0	73 (70.9)
Riboflavin (mg)	1.2	2.2	1.7–3.0	95 (92.2)
Niacin equivalents ^1^ (mg)	14	39.4	29.4–51.6	101 (98.1)
Vitamin C (mg)	38 (≤18 y) 40	184.5	113.9–239.6	97 (94.2)
Dietary folate equivalents ^2^ (μg)	520	563.9	416.4–672.3	59 (57.3)
Retinol equivalents ^3^ (μg)	530 (≤18 y) 550	1057	680.1–1418.2	86 (83.5)
Potassium (mg)	2800 *	3399.5	2695.3–4307.8	72 (69.9)
Magnesium (mg)	290 ^+^	358	285.9–422.6	75 (72.8)
Calcium (mg)	1050 (≤18 y) 840	843.7	557.3–1101.7	52 (50.5)
Phosphorus (mg)	1055 (≤18 y) 580	1447.6	1115.9–1903.8	98 (95.2)
Iron (mg)	23 (≤18 y) 22	10.7	7.7–13	4 (3.88)
Zinc (mg)	8.5 (≤18 y) 9.0	12.2	9.0–15.4	78 (75.7)
**Exceeding**	**Upper Limit**	**Median**	**IQR**	**Exceeding Upper Limit** ***n* (%)**
Sodium (mg)	460–920 *	1914.5	1434.2–2441.1	95 (92.2) ^4^
%E Saturated fat	10% ^5^	15	13–17	99 (96.1)

EAR: Estimated Average Requirement. AI: Adequate Intake. IQR: Interquartile range. Star (*) denotes adequate intake. ^+^ Magnesium recommendations are for pregnant women aged 19–30 years. The recommendation for pregnant women aged 31–50 years is EAR: 300mg/day. ^1^ Niacin intakes and requirements are expressed as niacin equivalents: 1 mg niacin equivalent = 1 mg niacin/60 mg tryptophan. Niacin + Niacin derived from tryptophan. ^2^ 1 µg dietary folate equivalent = 1 µg food folate = 0.5 µg folic acid on an empty stomach = 0.6 µg folic acid with meals or as fortified foods. ^3^ Retinol + (Beta-carotene/6) + (alpha-carotene/12) + (cryptoxanthin/12). ^4^ Comparison made relative to the upper end level of sodium AI. ^5^ For chronic disease risk reduction for general population.

**Table 3:** Australian Recommended Food Score (ARFS) for diet quality and daily energy intake from core and non-core foods in pregnant Indigenous women in the *Gomeroi gaaynggal* cohort (*n* = 103).

ARFS (Maximum Possible Score)	Median	IQR
Total (73)	28	21–3 5
Vegetables (21)	11	6–1 5
Fruit (12)	5	2–7
Meat (7)	2	1–3
Meat and alternatives (6)	1	0–1
Grains (13)	3	2–4
Dairy (11)	4	3–5
Water (1)	1	0–1
Condiments (2) *	1	1–2
**Other**	**Median**	**IQR**
Core foods ^1^ (kJ)	5414	3863–7180
Core foods (% Energy) ^1,2^	59	51–69
Non-core foods ^3^ (kJ)	3481	2236–4919
Non-core foods (% Energy) ^2,3^	41	31–49

IQR: Interquartile range. * Condiments include vegemite and tomato sauce for their nutritional properties. ^1^ Core food includes breads and cereals, fruit, vegetables, dairy and alternatives, meat and alternatives. ^2^ Calculated as a percentage of total daily energy intake. ^3^ Non-core foods include soft drinks, sweets and energy-dense nutrient-poor foods.

**Table 4:** Association between maternal dietary intake and fetal and birth weight.

	EFW (g) ^1^			Birth Weight (g) ^2^		
	n	Coefficient	95% CI	n	Coefficient	95% CI
**Micronutrients**						
Retinol equivalents (µg)	**96**	**−0.13**	**−0.21, −0.04**	**96**	**−0.24**	**−0.42, −0.06**
Dietary folate equivalents (ug)	96	−0.002	−0.24, 0.23	96	0.18	−0.29, 0.66
Iron (mg)	96	3.68	−9.61, 17.0	96	5.58	−21.2, 32.4
Sodium (mg)	96	−0.03	−0.1, 0.02	96	−0.02	−0.15, 0.1
Calcium (per 100 mg)	96	1.26	−10.9, 13.4	96	13.4	−11.2, 38
Zinc (mg)	96	2.61	−8.86, 14.07	96	6.11	−17.2, 29.44
**Macronutrients**						
Protein (g)	96	−0.34	−1.73, 1.05	96	−0.02	−2.86, 2.82
Carbohydrate (g)	96	−0.33	−0.81, 0.15	96	−0.45	−1.43, 0.52
Protein: Carbohydrate	96	340	−57.8, 739	96	469	−345, 1284
**Energy**						
Energy (KJ)	96	−0.01	−0.02, 0.01	96	−0.01	−0.04, 0.02
% Energy from core foods	**96**	**5.02**	**0.6, 9.45**	96	4.85	−4.38, 14.08
% Energy from non-core foods	**96**	**−5.02**	**−9.45, −0.6**	96	−4.85	−14.1, 4.38
**Diet quality**						
ARFS	96	−0.49	−6.16, 5.18	96	1.96	−9.71, 13.63

ARFS: Australian Recommended Food Score. EFW: estimated fetal weight. CI: confidence interval. ^1^ Adjusted for smoking, gestational age in days (at ultrasound), Pre-pregnancy Body Mass Index. ^2^ Adjusted for smoking, gestational age in days (at delivery), Pre-pregnancy Body Mass Index. Bold—*p*-value < 0.05.

**Table 5:** Association between maternal dietary intake and fetal kidney volume in the 3rd trimester.

	Combined Kidney Volume (cm^3^) ^1^			Combined Kidney Volume: EFW (cm^3^/kg) ^2^		
	*n*	Coefficient	95% CI	*n*	Coefficient	95% CI
**Micronutrients**						
Retinol equivalents (µg)	97	−0.0001	−0.002, 0.002	97	0.0003	−0.0004, 0.001
Dietary folate equivalents (ug)	97	−0.0003	−0.005, 0.004	97	0.0002	−0.002, 0.002
Iron (mg)	97	−0.15	−0.38, −0.07	97	−0.04	−0.14, 0.06
Sodium (mg)	97	−0.0003	−0.001, 0.001	97	−0.0001	−0.001, 0.0004
Calcium (per 100mg)	97	−0.07	−0.29, 0.15	97	−0.04	−0.13, 0.06
Zinc (mg)	97	−0.12	−0.32, 0.09	97	−0.05	−0.13, 0.04
**Macronutrients**						
Protein (g)	97	−0.01	−0.03, 0.02	97	−0.004	−0.01, 0.001
Carbohydrate (g)	97	−0.004	−0.01, 0.004	97	−0.001	−0.004, 0.003
Protein: Carbohydrate	97	−0.23	−7.5, 7.08	97	−1.55	−4.73, 1.62
**Energy**						
Energy (KJ)	97	−0.0001	−0.0003, 0.0001	97	−0.00003	−0.0001, 0.0001
% Energy from core foods	97	−0.03	−0.11, 0.05	97	−0.02	−0.06, 0.01
% Energy from non-core foods	97	0.03	−0.05, 0.1	97	0.02	−0.01, 0.06
**Diet quality**						
ARFS	97	−0.06	−0.16, 0.04	97	−0.02	−0.06, 0.03

ARFS: Australian Recommended Food Score. EFW: estimated fetal weight. CI: confidence interval. ^1^ Adjusted for EFW (kg) and smoking. ^2^ Adjusted for smoking.

**Table 6:** Association between maternal nutrient intake, including supplementation and fetal and birth weight.

	EFW (g) ^1^			Birth Weight (g) ^2^		
	*n*	Coefficient	95% CI	*n*	Coefficient	95% CI
**Micronutrients**						
Calcium (per 100 mg)	96	1.87	−10.2, 14	96	15.3	−9.14, 40
Folate equivalents (ug)	96	0.09	−0.03, 0.21	**96**	**0.33**	**0.09, 0.57**
Iron (mg)	96	2.31	−0.19, 4.81	**96**	**7.0**	**2.1**, **11.87**
Zinc (mg)	96	6.52	−2.77, 15.8	96	19.43	0.78, 38.1

EFW: estimated fetal weight. CI: confidence interval. ^1^ Adjusted for smoking, gestational age in days (at ultrasound), Pre-pregnancy Body Mass Index. ^2^ Adjusted for smoking, gestational age in days (at delivery), pre-pregnancy Body Mass Index. Bold—*p*-value < 0.05.

**Table 7:** Association between maternal nutrient intake, including supplementation and fetal kidney volume in the 3rd trimester.

	Combined Kidney Volume (cm^3^) ^1^			Combined Kidney Volume: EFW (cm^3^/kg) ^2^		
	*n*	Coefficient	95% CI	*n*	Coefficient	95% CI
**Micronutrients**						
Calcium (per 100 mg)	97	−0.07	−0.3, 0.14	97	−0.04	−0.14, 0.06
Folate equivalents (ug)	97	−0.001	−0.003, 0.001	97	−0.001	−0.002, 0.0003
Iron (mg)	97	−0.03	−0.08, 0.02	97	−0.02	−0.04, 0.001
Zinc (mg)	97	−0.13	−0.3, 0.04	97	−0.07	−0.15, 0.002

EFW: estimated fetal weight. CI: confidence interval. ^1^ Adjusted for EFW (kg) and smoking. ^2^ Adjusted for smoking.

## Data Availability

The data presented in this study are available on request from the corresponding author and the *Gomeroi gaaynggal* Advisory Committee. The data are not publicly available due to ethical reasons.

## Supplementary Materials

The following are available online at https://figshare.com/s/cb9bac30006cdc5ba4f7.
