# Supplementary material for: Maternal Diet Influences Fetal Growth but Not Fetal Kidney Volume in an Australian Indigenous Pregnancy Cohort
Source: Nutrients. 2021 Feb 9;13(2):569. doi: 10.3390/nu13020569 (PMC7914647; doi:10.3390/nu13020569)
Supplement: Supplementary file 1 [file nutrients-13-00569-s001.pdf]

nutrients

MDPI

Supplementary

Maternal Diet Influences Fetal Growth but Not Fetal Kidney Volume in an Australian Indigenous Pregnancy Cohort

Yu Qi Lee [1], Eugenie R Lumbers [2,3], Tracy L Schumacher [4,5,6], Clare E Collins [4,7], Kym M Rae [8,9], Kirsty G Pringle [2,3,*] and *Gomeroi gaaynggal* Advisory Committee

Table S1. Association between maternal dietary intake and fetal and birth weight.

	EFW (g)[1]			Birth Weight (g)[2]		
	n	Coefficient	95% CI	n	Coefficient	95% CI
			Micronutrients			
Thiamin (mg)	96	10.9	−70.8, 92.5	96	51.2	−113, 215
Riboflavin (mg)	96	−13.6	−68.3, 41.2	96	14.7	−96.9, 126
Niacin eq (mg)	96	−2.53	−8.73, 3.67	96	−1.01	−13.7, 11.7
Vitamin C (mg)	96	−0.18	−0.63, 0.27	96	−0.04	−0.97, 0.9
Magnesium (mg)	96	−0.07	−0.54, 0.4	96	0.1	−0.85, 1.05
Phosphorus (mg)	96	−0.02	−0.11, 0.07	96	0.02	−0.16, 0.2
			Macronutrients			
Total Fat (g)	96	−0.59	−1.98, 0.79	96	−0.28	−3.1, 2.55
Sat Fat (g)	96	−1.12	−4.07, 1.83	96	−0.1	−6.13, 5.92

EFW: estimated fetal weight. CI: confidence interval.
[1] Adjusted for smoking, gestational age in days (at ultrasound), Pre-pregnancy Body Mass Index.
[2] Adjusted for smoking, gestational age in days (at delivery), Pre-pregnancy Body Mass Index.

Table S2. Association between maternal dietary intake and fetal kidney volume in the 3rd trimester.

	Combined kidney volume (cm^3) [1]			Combined kidney volume: EFW (cm^3/kg) [2]		
	n	Coefficient	95% CI	n	Coefficient	95% CI
			Micronutrients			
Thiamin (mg)	97	−0.57	−2.0, 0.86	97	−0.08	−0.7, 0.56
Riboflavin (mg)	97	−0.56	−1.53, 0.41	97	−0.2	−0.63, 0.23
Niacin eq (mg)	97	−0.02	−0.08, 0.04	97	−0.01	−0.06, 0.04
Vitamin C (mg)	97	0.002	−0.01, 0.01	97	0.002	−0.002, 0.005
Magnesium (mg)	97	−0.004	−0.01,0.004	97	−0.001	−0.005, 0.002
Phosphorus (mg)	97	−0.001	−0.002, 0.001	97	−0.0002	−0.001, 0.001
			Macronutrients			
Total Fat (g)	97	−0.01	−0.03, 0.01	97	−0.003	−0.01, 0.01
Sat Fat (g)	97	−0.02	−0.07, 0.03	97	−0.01	−0.03, 0.02

EFW: estimated fetal weight. CI: confidence interval.
[1] Adjusted for EFW (kg) and smoking.
[2] Adjusted for smoking.
